# Case Report: Thrombosed Mitral Leaflets in a Patient With Rheumatic Heart Disease: A Rare Clinical Case

**DOI:** 10.1002/ccr3.72082

**Published:** 2026-02-16

**Authors:** Naser Khezerlou, Mehdi Taghizadeh, Mohammadjavad Sotoudeheian, Razieh Parizad

**Affiliations:** ^1^ Cardiovascular Research Center Tabriz University of Medical Science Tabriz Iran; ^2^ Physiology Research Center Iran University of Medical Science Tehran Iran

**Keywords:** anticoagulation, mitral valve thrombus, rheumatic mitral stenosis, stroke, transesophageal echocardiography

## Abstract

Rheumatic heart disease remains a significant contributor to cardiovascular morbidity. We report the case of an 82‐year‐old man who presented with acute right‐sided hemiplegia. Transesophageal echocardiography (TEE) showed a thrombus attached to the mitral valve leaflets (MVL). Anticoagulation therapy was initiated, leading to significant thrombus resolution. It spotlights the potential efficacy of thrombolytic and anticoagulation.

**Committee Approval Number:** IR.TBZMED.REC.1403.1018

**Abbreviations**
2D: two‐dimensional
3D: three‐dimensional
AF: atrial fibrillation
BCs: blood cultures
CHF: congestive heart failure
CRP: C‐reactive protein
CT: computed tomography
EF: ejection fraction
EKG: electrocardiography
ESR: erythrocyte sedimentation rate
HF: heart failure
ICU: intensive care unit
INR: international normalized ratio
MR: mitral regurgitation
MRI: magnetic resonance imaging
MS: mitral stenosis
MV: mitral valve
MVA: mitral valve area
MVL: mitral valve leaflets
MVT: mitral valve thrombosis
RHD: rheumatic heart disease
RMS: rheumatic mitral stenosis
TEE: transesophageal echocardiography
tPA: tissue plasminogen activator
TTE: transthoracic echocardiography
WBC: white blood cell count

## 1 Introduction

Rheumatic heart disease (RHD) remains a major cause of cardiovascular morbidity and mortality, particularly in low‐ and middle‐income countries [1, 2]. It commonly affects the mitral valve (MV), leading to complications such as mitral stenosis (MS) and regurgitation [3]. Although these sequelae are well‐documented, thrombosis of the mitral valve leaflets (MVL) is a rare but potentially life‐threatening complication that may result in acute heart failure (HF) or systemic embolization [4, 5]. The exact mechanism of thrombus formation in RHD remains unclear; however, it is hypothesized that endothelial damage, blood stasis, and, hypercoagulability collectively contribute to Virchow's triad [6].

Several recent case reports have described instances of native MV thrombosis in patients with RHD. For instance, a case study [7] reported a case of a 71‐year‐old woman with rheumatic MS who developed a thrombus on her native MV despite being on anticoagulation therapy. Similarly, Collings et al. [8] presented a case in which a patient with long‐standing MS developed a spontaneous obstructive thrombus, necessitating emergency valve replacement. These cases illustrate the critical role of detailed cardiac investigation.

Advanced imaging techniques, notably transesophageal echocardiography (TEE), play a crucial role in the early detection of valvular thrombosis [9]. However, the optimal management strategy remains controversial, with treatment options, including anticoagulation, thrombolysis, or surgical intervention, depending on the severity of obstruction and embolic risk [10, 11]. Given the rarity and potentially fatal prognosis of this condition, maintaining a high index of clinical suspicion and making prompt therapeutic decisions are crucial for improving patient outcomes.

## 2 Case History/Examination

An 82‐year‐old male with a history of smoking presented to our hospital's emergency department with dysarthria and sudden‐onset right‐sided hemiplegia. His medical history consisted of hypertension, benign prostatic hyperplasia, Parkinson's disease, and a previous cerebrovascular accident without residual deficits. His current medications included atorvastatin, levodopa, pantoprazole, finasteride, and amitriptyline.

Upon initial evaluation, the patient's vital signs were as follows: pulse rate of 80 beats/min, blood pressure of 165/100 mmHg, respiratory rate of 28 breaths/min, oxygen saturation of 82% on room air, and a body temperature of 36.5°C.

Neurological examination revealed right‐sided facial weakness graded as 1/5. Muscle tone was increased in the right limbs but normal in the left. Muscle strength was 1/5 in the right limbs and 5/5 in the left. Abnormal findings comprised a positive extensor plantar reflex (Babinski sign) on the right side.

Cardiac auscultation demonstrated a Grade III/VI harsh systolic murmur, best heard at the apex. Pulmonary examination indicated expiratory wheezes and bilateral fine crepitations. Additionally, erythematous papules and skin coarsening were noted. The rest of the peripheral examination was unremarkable.

## 3 Differential Diagnosis, Investigations and Treatment

A non‐contrast computed tomography (CT) scan of the brain detected chronic microangiopathic changes and age‐related parenchymal volume loss. Due to a high clinical suspicion of ischemic stroke (thrombotic), the patient received intravenous tissue plasminogen activator (tPA) and was subsequently transferred to the intensive care unit (ICU) for close monitoring and further management.

The patient was started on therapeutic anticoagulation with intravenous heparin infusion alongside with warfarin tablet. The decision not to perform surgery and to choose drug treatment alone was made due to the patient's preference, economic situation, and the high risk of surgery due to his advanced age and concomitant diseases. A repeat brain CT scan 24 h after the initial scan confirmed a hypodense lesion in the frontoparietal lobe, indicative of an ischemic stroke, without signs of parenchymal hemorrhage (Figure 3B: showing a hypodense area in the frontoparietal lobe, consistent with ischemic stroke).

**FIGURE 1:**
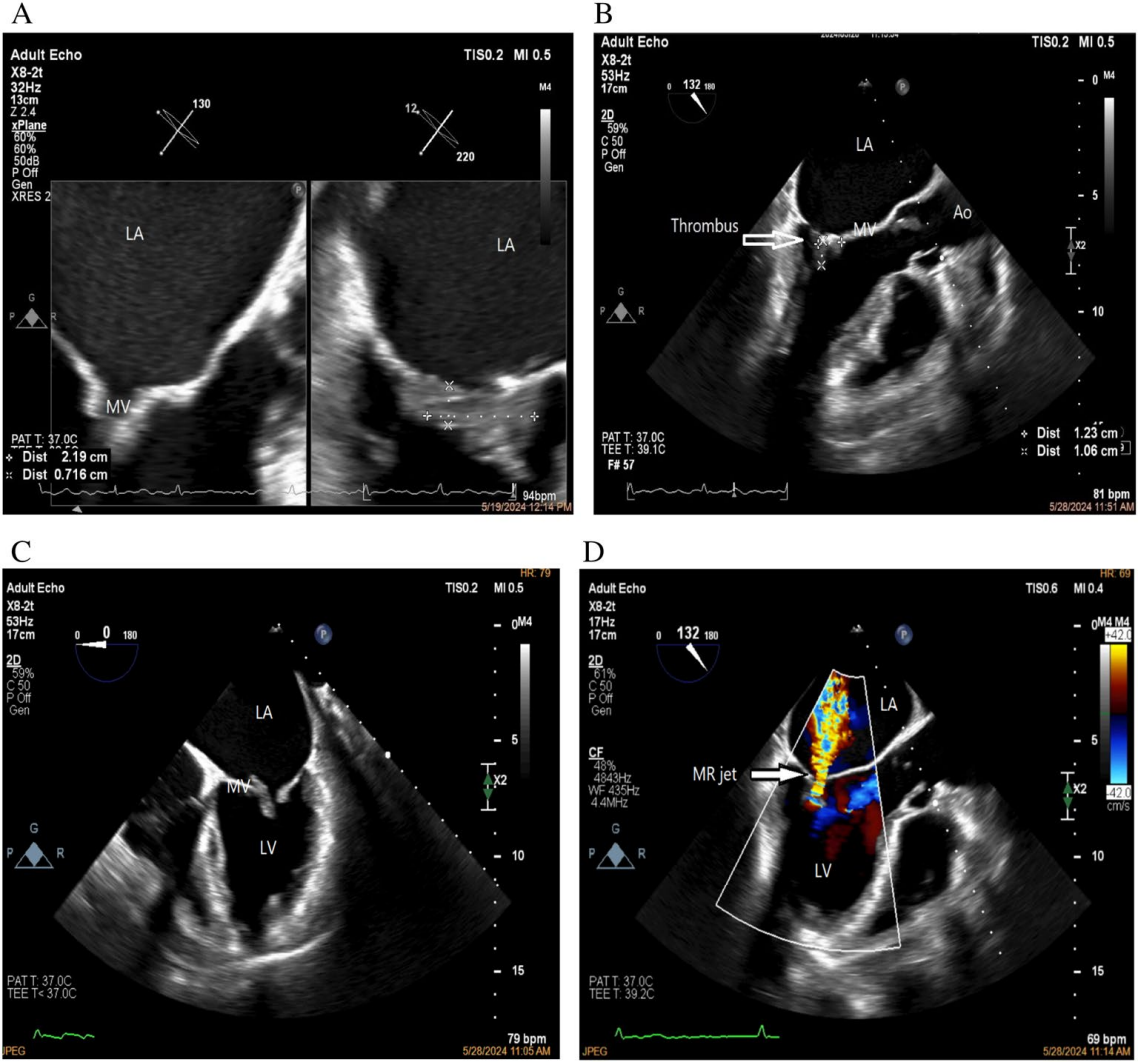
(A) Two‐dimensional transesophageal echocardiography (TEE) showing a hypoechoic mass around the leaflet tips, suggestive of thrombus or vegetation (size = 2.19 × 0.71 cm). (B) Two‐dimensional transesophageal echocardiography (TEE). Control echocardiography after 1 week of admission revealed a significant decrease in the size of the mass (size = 1.23 × 1.06 cm). (C) Two‐dimensional transesophageal echocardiography (TEE), esophageal view, showing mitral valve opening in mid‐diastole. A hypoechoic mass was observed on the anterior mitral valve leaflet (AMVL), and opening of the MV was restricted. (D) Two‐dimensional transesophageal echocardiography (TEE). Colored doppler shows a moderate regurgitant flow of mitral valve (MV).

Electrocardiography (EKG) showed atrial fibrillation (AF). A subsequent TTE revealed that the left ventricle was of normal size but exhibited mildly reduced systolic function, with the ejection fraction (EF) visually estimated at approximately 35%. The MV appeared thickened and dome‐shaped, with significant calcification at the medial commissure, findings consistent with RHD. Severe MS and moderate mitral regurgitation (MR) (Figure 1D: colored doppler showing a moderate regurgitant flow of mitral valve [MV]) were present. Furthermore, a hypoechoic fixed mass was identified at the tips of the mitral leaflets, particularly between A2‐P2 and A3‐P3, raising suspicion of either a thrombus or vegetation (Movies S1, S2: TEE showing thickened and dome‐shaped MV, with significant calcification with a hypoechoic mass at the tips of the mitral leaflets, particularly between A2 and P2.) (Figure 1A showing a hypoechoic mass around the leaflet tips, suggestive of thrombus or vegetation) (Figure 1C: showing mitral valve opening in mid‐diastole. A hypoechoic mass on the anterior mitral valve leaflet). The mitral valve area (MVA) was measured at 1.45 cm^2^ using both two‐dimensional (2D) and three‐dimensional (3D) planimetrics, with the mean pressure gradient estimated at 4 mmHg at a heart rate of 85 beats/min (low gradient due to the simultaneous presence of systolic and diastolic failure and high left ventricular filling pressure and higher afterload).

Laboratory investigations identified mildly elevated inflammatory markers, including an erythrocyte sedimentation rate (ESR) of 36 mm/h (reference range: 0–15 mm/h) and a C‐reactive protein (CRP) level of 69 mg/dL (reference: < 0.5 mg/dL). The white blood cell count (WBC) was 5900/mm^3^, and serum creatinine was 1.34 mg/dL. Rheumatoid factor was within the normal range. Blood cultures (BCs), collected in two separate sets, showed no bacterial growth.

**FIGURE 2:**
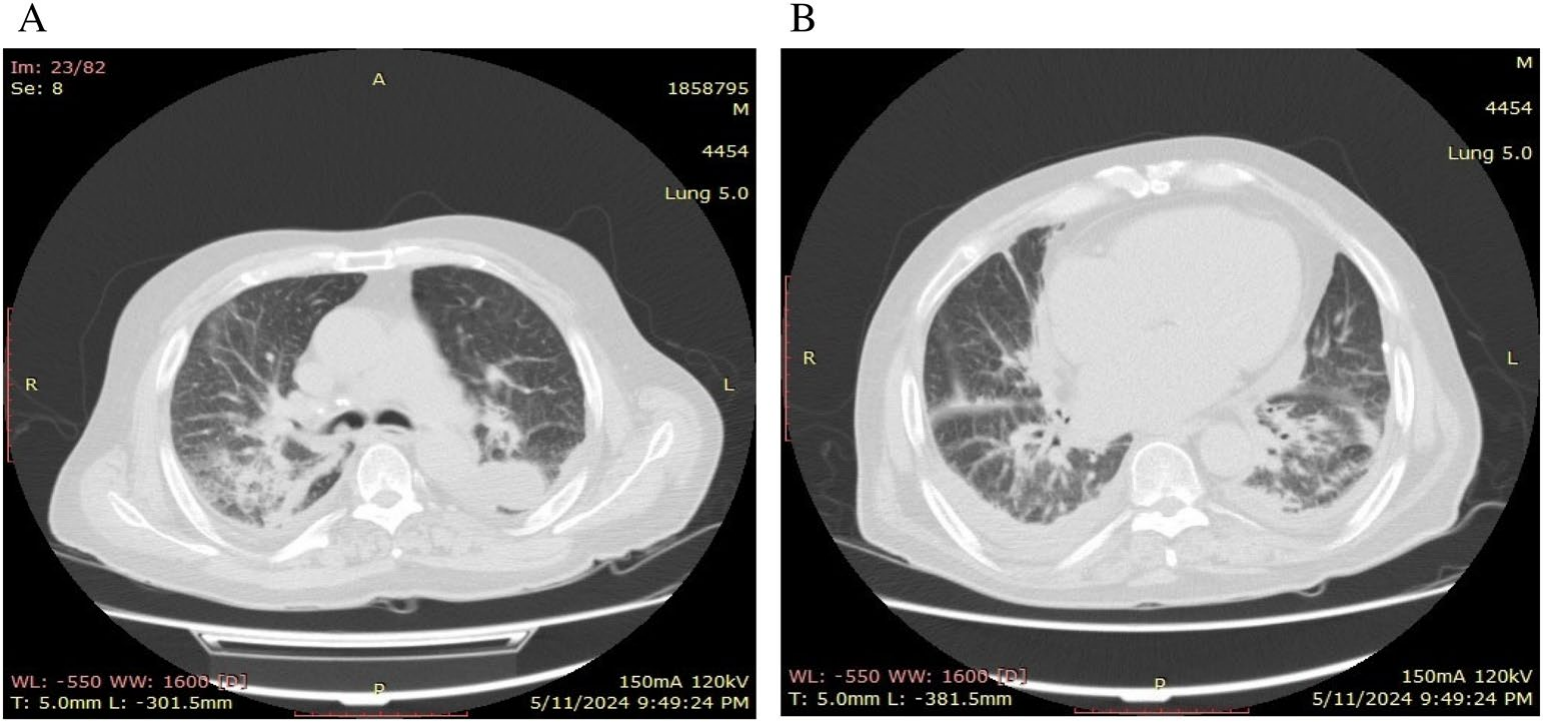
(A) A chest computed tomography scan (CT scan) demonstrated multiple calcified mediastinal and hilar nodules, calcified granulomas in both lungs, and air‐space opacification. (B) A chest computed tomography scan (CT scan) demonstrated an increase in the thickness of the interlobular septum, bilateral pleural effusions, and accumulation of fluid in the fissure of both sides.

A chest CT scan displayed thickened interlobular septa, bilateral pleural effusions, fluid accumulation within the fissures of both lungs, multiple calcified mediastinal and hilar nodules, and calcified granulomas in both lungs (Figure 2A: multiple calcified mediastinal and hilar nodules, calcified granulomas in both lungs and air‐space opacification). Moreover, air‐space opacification suggested concurrent pneumonia and congestive heart failure (CHF) (Figure 2B: increase in the thickness of the interlobular septum, bilateral pleural effusions, and accumulation of fluid in the fissure of both sides).

**FIGURE 3:**
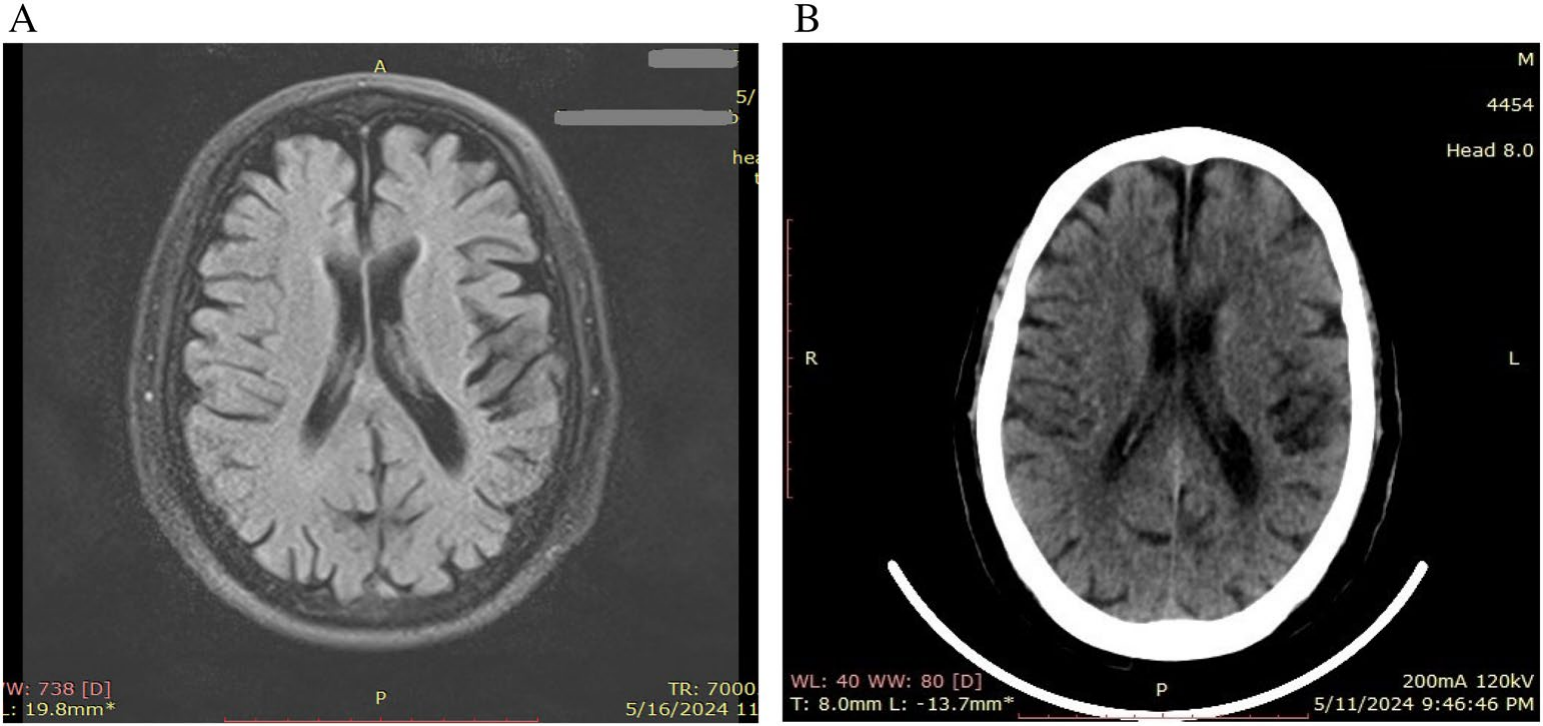
(A) Magnetic resonance imaging (MRI) of the head that was performed 7 days after admission shows gliosis and malacia at left frontoparietal lobes and cortical focus in the superior gyrus of the left frontal lobe in favor of acute ischemia. (B) Follow‐up brain computed tomography (CT) scan shows a hypodense area in the frontoparietal lobe, consistent with ischemic stroke and no evidence of intracranial hemorrhage.

A brain magnetic resonance imaging (MRI) scan performed 7 days after admission revealed gliosis and malacia in the left frontoparietal lobe, along with a cortical ischemic focus in the superior gyrus of the left frontal lobe, confirming acute ischemia (Figure 3A: gliosis and malacia at left frontoparietal lobes and cortical focus in the superior gyrus of the left frontal lobe).

A follow‐up echocardiogram conducted 1 week later showed a significant reduction in the size of the hypoechoic mass on the MV, strongly suggesting thrombus resolution with ongoing anticoagulation therapy (Figure 1B: Control echocardiography after 1 week of admission revealed a significant decrease in the size of the mass). The Duke criteria were not met. No clinical or laboratory evidence of infective endocarditis was observed, and blood cultures remained negative throughout hospitalization.

## 4 Followed Up and Outcomes

The patient was followed up in the 2th month and the 9th month after discharge. At the time of follow‐up, he showed significant improvement in neurological function, with muscle strength in the right limbs increasing to 4/5. He remained on therapeutic anticoagulation with warfarin, and his international normalized ratio (INR) was within the target range (2.0–3.0). No further embolic events were reported, and the patient denied any new symptoms of heart failure or stroke. He was able to perform daily activities with minimal assistance.

## 5 Conclusion

In conclusion, this case illustrated contributes to the growing body of literature on thrombosed mitral valve leaflets in patients with RHD. It spotlights the critical role of advanced imaging techniques in early diagnosis and the potential efficacy of anticoagulation therapy in selected cases. The findings stress the importance of maintaining a high index of suspicion for valvular thrombosis in RHD patients, especially those with AF and MS. Future research should focus on optimizing diagnostic and therapeutic strategies for this rare but potentially life‐threatening complication, emphasizing individualized patient care and multidisciplinary collaboration.

## 6 Discussion

Thrombosis of native MVL in the setting of RHD is an exceptionally rare occurrence with significant clinical implications. While MS is a well‐recognized sequela of RHD, thrombus formation on the MVL without concomitant infective endocarditis remains an underreported phenomenon. This case reports the importance of a comprehensive cardiac evaluation in stroke patients, particularly those with predisposing valvular pathology.

Several pathophysiological mechanisms have been proposed to explain mitral valve thrombosis (MVT) in RHD. The persistent inflammation and fibrotic changes characteristic of RHD contribute to endothelial dysfunction, which, in conjunction with blood stasis due to MS and a hypercoagulable state, fulfills Virchow's triad for thrombus formation [2, 6]. AF, which is frequently associated with MS, further exacerbates the thromboembolic risk by promoting left atrial stasis and clot formation [5]. This patient's history of AF and prior cerebrovascular events suggests a heightened susceptibility to cardioembolic complications.

Management of MVT is controversial and often individualized based on the severity of obstruction, embolic risk, and hemodynamic compromise. Anticoagulation remains the mainstay of therapy, with warfarin and heparin frequently employed to promote thrombus resolution and prevent further embolic events [11]. Thrombolytic therapy has been explored in cases of prosthetic valve thrombosis, but its role in native MVT is not well established due to concerns regarding bleeding risks and potential embolization [10]. In cases where thrombus burden is significant and leads to severe valve obstruction, surgical intervention, including thrombectomy or valve replacement, may be required [8]. Fortunately, in this case, the patient exhibited significant thrombus regression with anticoagulation, obviating the need for surgical management.

The implications of this case extend beyond individual patient management, underlining the necessity for increased awareness and vigilance in identifying rare thrombotic complications of RHD. Given the global burden of RHD, particularly in low‐ and middle‐income countries, routine screening with echocardiography in high‐risk populations may aid in early detection and intervention [3].

## Author Contributions

**Naser Khezerlou:** resources, supervision. **Mehdi Taghizadeh:** writing – review and editing. **Mohammadjavad Sotoudeheian:** writing – review and editing. **Razieh Parizad:** software, writing – review and editing.

## Funding

The authors have nothing to report.

## Consent

This case‐report study was conducted after obtaining informed consent from the patient upon admission to the teaching and research hospital, and upon receiving ethical approval from the university's ethics committee. The case contains no patient‐identifying information.

## Supporting information


**Movie S1:** Two‐dimensional transesophageal echocardiography (TEE) showing thickened and dome‐shaped MV, with significant calcification with a hypoechoic mass at the tips of the mitral leaflets, particularly between A2 and P2.

**Movie S2:** Two‐dimensional transesophageal echocardiography (TEE) showing thickened and dome‐shaped MV, with significant calcification with a hypoechoic mass at the tips of the mitral leaflets, particularly between A2 and P2.

## Data Availability

All data used or generated in this study are included in this article. Further information is available from the corresponding author upon request.
